# Towards an atomistic understanding of disordered carbon electrode materials†
†Electronic supplementary information (ESI) available: Computational details; further analysis; coordinate files in various formats. See DOI: 10.1039/c8cc01388h


**DOI:** 10.1039/c8cc01388h

**Published:** 2018-05-23

**Authors:** Volker L. Deringer, Céline Merlet, Yuchen Hu, Tae Hoon Lee, John A. Kattirtzi, Oliver Pecher, Gábor Csányi, Stephen R. Elliott, Clare P. Grey

**Affiliations:** a Department of Engineering , University of Cambridge , Cambridge CB2 1PZ , UK . Email: vld24@cam.ac.uk; b Department of Chemistry , University of Cambridge , Cambridge CB2 1EW , UK . Email: cpg27@cam.ac.uk; c CIRIMAT , Université Toulouse 3 Paul Sabatier , CNRS, INPT , Bât. CIRIMAT , 118, route de Narbonne , Toulouse cedex 9 31062 , France; d Réseau sur le Stockage Électrochimique de l'Énergie (RS2E) , Fédération de Recherche CNRS 3459 , Amiens 80039 , France; e College of Chemistry and Chemical Engineering , Xiamen University , Xiamen 361005 , People's Republic of China; f NMR Service GmbH , Blumenstr. 70 Haus 3 , Erfurt 99092 , Germany

## Abstract

Disordered nanoporous and “hard” carbons are widely used in batteries and supercapacitors, but their atomic structures are poorly determined. Here, we combine machine learning and DFT to obtain new atomistic insight into carbonaceous energy materials. We study structural models of porous and graphitic carbons, and Na intercalation as relevant for sodium-ion batteries.

## Abstract 2

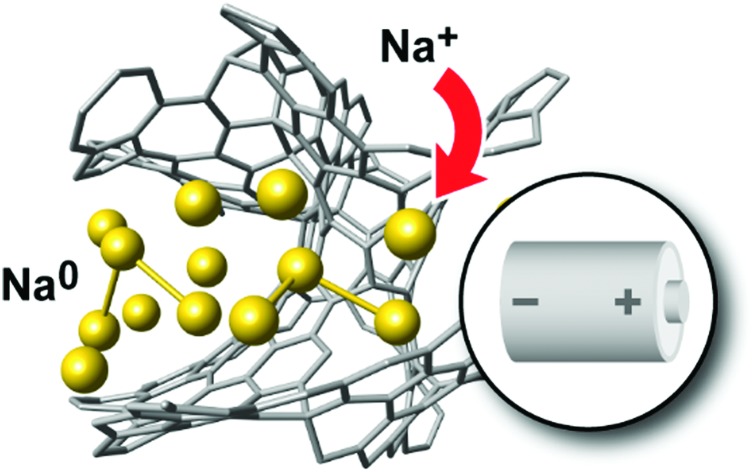
Machine-learning and DFT modelling, linked to experimental knowledge, yield new insight into the structures and reactivity of carbonaceous energy materials.

Nanostructured forms of elemental carbon are widely used as powerful, generally non-toxic, and economic electrode materials in Li-ion and Na-ion batteries and supercapacitors, while also being employed to ensure electrical contact between particles within battery electrodes and in filtration.^1^–^5^ Structurally, these materials are intermediate between crystalline and amorphous states, exhibiting locally graphitic-like fragments but no long-range order beyond a few nanometres (Fig. 1). Many carbons contain hierarchical (nano-, meso-, and macroscale) porosity, the nature of the pores and their connectivity being critical for device performance. The details of their atomistic structures are diverse and far from being fully known.

**Fig. 1:**
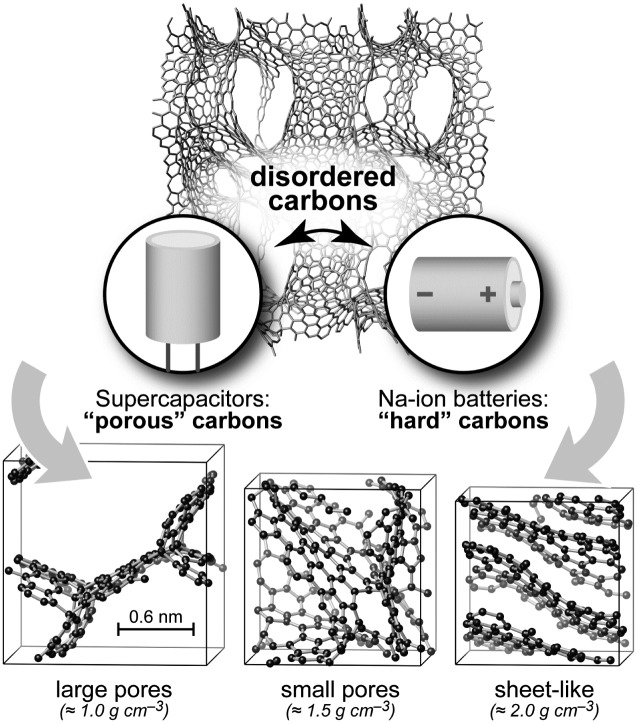
Structural models of disordered carbons and their most relevant applications. Top: Example 930-atom structure (≈1 g cm^–3^), created in a long GAP-driven MD simulation. A 2 × 2 × 1 simulation-box expansion is shown to make the pore structure more visible. Bottom: Smaller structural models, containing ≈200 atoms, drawn using VESTA.^21^ On the left, a scale bar shows the experimentally determined average pore diameter in TiC-CDC-600 samples (see below); larger pore sizes are seen experimentally in samples prepared at higher temperature.^22^ These figures provide only three examples of the pore structures generated in this work and a more detailed discussion of pore sizes is provided in the ESI.†

Important pieces of the puzzle have been added by local probes, such as NMR, Raman, and electron energy-loss spectroscopy (EELS),^6^–^9^ by transmission electron microscopy (TEM),^10^,^11^ and by pair distribution function (PDF) and Reverse Monte Carlo (RMC) modelling of diffraction data; the latter can be coupled to interatomic potentials (“hybrid RMC”).^12^–^14^ To complement experiments, molecular-dynamics (MD) simulations are increasingly used to create structures by quenching from the melt^15^–^17^ or annealing disordered precursors.^18^–^20^

Despite their usefulness, atomistic simulations of disordered carbons suffer from a severe trade-off between accuracy and speed. Quantum-mechanical methods, such as density-functional theory (DFT), provide accurate structures, but are too computationally expensive for the large system sizes required. By contrast, classical empirical potentials often cannot fully describe the very diverse local environments and bonding mechanisms in disordered carbons: even various state-of-the-art empirical potentials may generate vastly different structures.^20^

In this Communication, we describe an approach that can provide this missing link between accuracy and speed, and thereby yield new microscopic insight into carbonaceous energy materials. We combine a machine-learning (ML)-based interatomic potential^23^,^24^ with DFT electronic-structure analyses and show how all this can be linked to experimental knowledge in the field. One goal is to generate various structural models with different system sizes and densities, with which to explore atomic and electronic structures of carbon frameworks—and the effect of these on a specific property, illustrated here for the case of Na intercalation. Subsequently, and hierarchically, using ML and quantum mechanics, our study provides proof-of-concept for a more general modelling strategy for energy materials.

We start by modelling nanoporous carbons as used in supercapacitors. We use our Gaussian approximation potential (GAP) for carbon,^24^ which has been “trained” on DFT data, being fitted to energies and forces for amorphous and partly graphitised configurations as well as bulk graphite. The potential itself is not modified during this study. We generated amorphous carbon (a-C) structural models at densities between 1.0 and 2.0 g cm^–3^ by rapid quenching from the melt. These precursors were then further annealed to form extended graphitic fragments (as shown before with empirical potentials; ref. 18–20).

We tested the accuracy of our GAP specifically for snapshots from such annealing trajectories: it achieves an energy accuracy to within 2 kJ mol^–1^ of DFT data (Fig. 2a) but completes the task several orders of magnitude faster. After 100 and 200 ps of simulation time, we remove any long carbon chains (···C–C≡C–C··· and longer) and atoms with only one neighbour (where they occur); these structural defects in actual samples are prone to oxidation at the elevated temperatures used to anneal/activate disordered carbons (being removed as CO), and thus are not expected to be found in the final samples. After annealing, the structures are further optimised and finally relaxed using dispersion-corrected DFT.^25^–^28^ Computational details are in the ESI.†

**Fig. 2:**
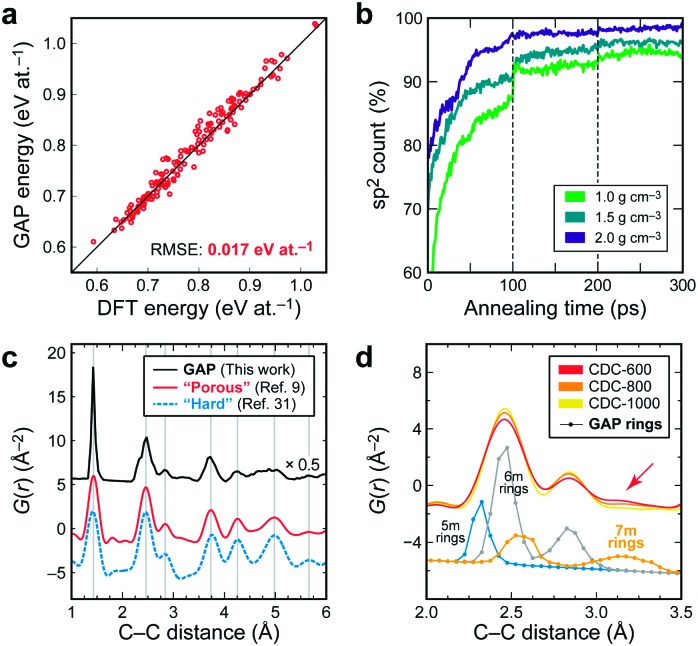
(a) DFT- *versus* GAP-computed energies for structures at various points of annealing trajectories. The root-mean-square error (RMSE) between these quantities is given. (b) Count of sp^2^-bonded atoms during annealing; dashed lines indicate removal of unphysical long chains (see text). (c) PDF analysis, comparing calculated results for the structure shown at the top of Fig. 1 (“GAP”, 930 atoms), to experimental data for a “porous” (CDC-600)^9^ and a “hard” carbon^31^ at room temperature, with arbitrary vertical offsets. The PDF for the GAP structure shows a sharp first peak, and thus has been scaled to ease visualisation. Vertical lines are guides to the eye. (d) Close-up of the PDF for CDCs after annealing at different temperatures (with progressive ordering),^9^ and calculated contributions to GAP structures from 5/6/7-membered rings individually (see ESI†). Experimental data reproduced with permission from ref. 9 and 31.

The most straightforward structural fingerprint of carbons is their atomic coordination relating to the local bonding (“sp/sp^2^/sp^3^”). The sp^2^ count in our model systems quickly rises during annealing (Fig. 2b), which agrees well with EELS experiments: in so-called carbide-derived carbons (CDCs), obtained by etching titanium out of a TiC matrix, the sp^2^ content is mostly >90% and increases with synthesis temperature.^7^,^16^,^29^ We compare a calculated PDF to representative experiments and find that it reproduces all general features (Fig. 2c); see also the ESI.†

A key piece of structural insight is given by ring statistics: in graphite, all rings are six-membered, but disorder can change this. In our structures, roughly every second ring is six-membered, and 5-/7-membered ones account for almost all the rest, largely independent of the density. While similar observations were recently made using one empirical potential,^19^ an earlier study found much larger counts of 6-membered rings, and no 5-membered ones at all.^15^ Although it is currently extremely difficult to quantify ring statistics experimentally, TEM images indeed proved the existence of 5-/7-membered rings in disordered carbons,^10^,^11^ and the presence of bent, “fullerene-like” fragments containing 5-membered rings has been suggested.^8^ Odd rings have been experimentally realised in “amorphous graphene”.^11^,^30^ Finally, the presence of 7-membered rings is suggested by an additional PDF contribution between 3.0 and 3.4 Å,^9^ likewise seen in our simulations (arrow in Fig. 2d).

Recent studies suggest that structural ordering in modelled graphitised carbons can be directly controlled by adjusting the annealing temperature.^20^ Accordingly, but beyond the scope of this initial Communication, we are planning to build a much larger library of structures generated using GAP-MD at various temperatures (and thus with various degrees of ordering). Among our long-term goals will be to use these libraries for the computer-based design of supercapacitor electrodes with optimized pore sizes and structure, and to develop direct links to local experimental probes such as NMR further.^32^–^34^

Here, instead, we highlight another aspect of our general strategy. Since we focus on relatively small structures, these are directly amenable to subsequent first-principles studies: once GAP has done the “heavy lifting”, the annealed structures serve as input for DFT. Thereby, we overcome two inherent and fundamental limitations of ML potentials. First, they give access to the atomic potential-energy surface but not to the electronic structure. Second, adding other species (such as Li or Na) to an ML potential requires a significant extension of the training database and often new technical developments.^35^ Both problems are circumvented by using DFT for these tasks instead.

We illustrate this by exploring the effect of Na insertion in disordered carbons, which currently represent the most promising anodes for Na-ion batteries. Na does not intercalate into graphite (the anode in commercial Li-ion batteries), but it does intercalate readily into disordered hard (“non-graphitisable”) carbons, with capacities approaching that seen for Li/graphite.^36^–^38^ More complex carbons from precursors such as chemically modified pitch^39^ or “soft” carbons from synthetic molecular precursors^40^–^42^ likewise intercalate Na. *In situ*^23^Na NMR and PDF measurements have recently been used by some of us to explore the intercalation mechanism in hard carbons.^31^

One key question concerns the energetics of intercalation. While it is straightforward to simulate ion adsorption on pristine (or defective) graphene, this is much more complicated in amorphous systems. Herein, we study a highly disordered “porous” structure containing 206 carbon atoms (density ≈1.4 g cm^–3^) as one example. In the future, these strategies will be straightforward to extend to more strongly graphitised and layered carbons (*cf.*Fig. 1). We begin by randomly placing single Na atoms in this cell, thus generating an ensemble of candidate adsorption sites, and optimise each candidate structure using DFT. This is in the spirit of *ab initio* random structure searching (AIRSS).^43^ The binding of Na is clearly favourable (Fig. 3a): adsorption energies on most sites range from –0.4 to –1.0 eV (grey; 0.4–1.0 V *vs.* Na metal). The Na environment for the point shown in red (at –1.6 eV) is close to both a 7-membered ring and a defect (a 2-coordinate carbon atom); the latter will likely be passivated (by hydrogenation or oxidation) during sample preparation or battery applications, before any Na enters the system. It is therefore not expected to be relevant for device performance.

**Fig. 3:**
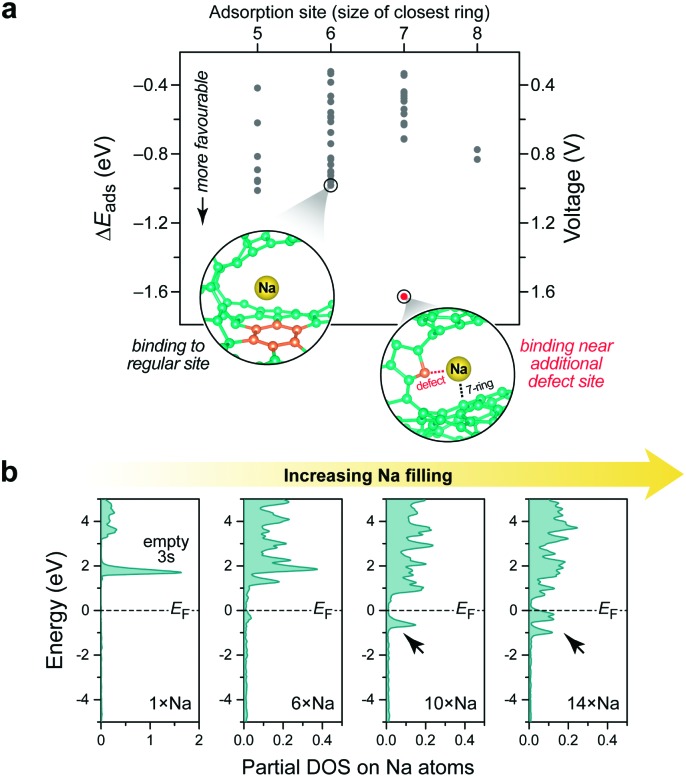
Modelling Na intercalation in a carbonaceous anode material. (a) Output of a stochastic search as described in the text. Two relevant atomic environments are visualised. (b) Electronic partial densities of states (DOS), comparing different systems with increasing Na intercalation (generated by DFT-MD annealing as described in the text).

While these AIRSS-like simulations sample many possible adsorption sites, it is furthermore possible to generate configurations by DFT-driven MD. We filled the systems with 6, 10, or 14 Na atoms (3–7 atom%), heated them using DFT-MD and subsequently quenched into local minima, leaving the carbon structure largely unaffected (ESI†). This readily led to Na intercalation in the large pore of the candidate structure, but not in a smaller one. We therefore probed different fillings in the same host structure and computed the partial electronic density of states (PDOS) at each stage (Fig. 3). Initially, a single inserted Na transfers its valence charge to the carbon framework completely, forming Na^+^, and the Na 3s orbital remains unoccupied above the Fermi level *E*_F_. With increasing filling, occupied Na levels occur—first with a zero, then with a finite partial DOS directly at *E*_F_ (arrows).

A closer look at the case with largest filling (Fig. 4) reveals distinct differences between individual Na sites. Indeed, lower Na charges (interpreted as resulting from electron back-transfer) are observed with increasing occurrence of Na–Na contacts in the nearest-neighbour shell. The same is reflected in the partial DOS (Fig. 4) as a complementary computational approach.

**Fig. 4:**
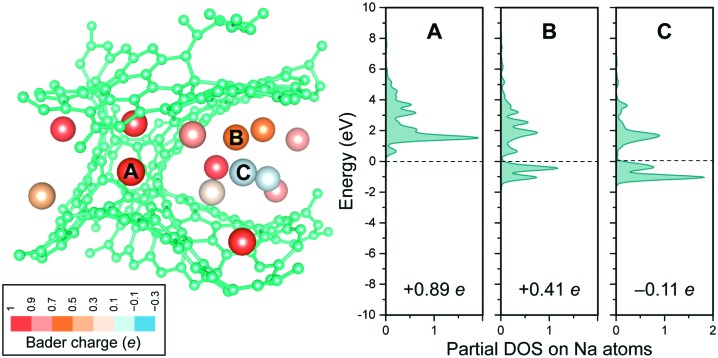
More detailed, atom-resolved insights into Na intercalation. Left: Optimised Na_14_C_206_ structure after DFT-MD annealing and cooling. Atoms are coloured according to their charge state (computed using Bader analysis).^44^ Right: Partial DOS but now for three individual, representative atoms, as marked, and their charges,^44^ both qualitatively indicating a gradual transition from Na^+^ to Na^0^. The slightly negative charge for atom C is within the expected deviation of the particular charge-partitioning scheme used.

These results are now compared with previous experimental observations, a sloping profile from ≈1.2 to 0.1 V, followed by a flatter region at ≈0.1–0.0 V *vs.* Na, being observed electrochemically and resulting in a total capacity of 250–400 mA h g^–1^ (NaC_9_–NaC_6_) depending on the nature of the carbon.^36^–^38^ The calculated voltages associated with the insertion of single atoms (Fig. 3a) are consistent with the sloping region, and the observation of Na^+^ cations (Fig. 3b) is consistent with “diamagnetic” ions seen by NMR.^31^ The NMR results were interpreted in terms of very distinct electronic structures for the Na atoms in the sloping and flatter regions, the former being associated with more localised electrons, the second with “metallic” behaviour and increased Na PDOS at *E*_F_, with increasing depth of discharge (measured *via* the Knight shift). Our calculations at a composition of NaC_15_ (≈160 mA h g^–1^) show a range of partial DOS values at *E*_F_ and charges on Na atoms (Fig. 4), consistent with NMR results at a similar composition:^31^ that is, at a state of charge where a transition from localised to metallic behaviour is occurring. More calculations are in progress to explore different carbon structures with different degrees of ordering (that can be partly controlled through the annealing protocol; *cf.*ref. 20), different counts of odd-membered rings (*cf.*Fig. 2d and 3a), and the effect of adding more Na on the atomic and electronic structure. Ultimately, this is expected to enable the computation and analysis of complete voltage profiles up to close to NaC_6_.

In conclusion, we have shown initial examples of how a combination of machine-learning and DFT modelling can provide new insight into disordered carbons for supercapacitor and battery electrode applications. Together with local experimental probes, previously used to study both the structure of porous carbons^9^ and the Na intercalation,^31^ this completes a tool-kit of complementary experimental and computational techniques for developing next-generation energy-storage materials.

We thank Dr Phoebe Allan, Dr Matt Cliffe, and Dr Rachel Kerber for useful discussions. V. L. D. acknowledges a Feodor Lynen Fellowship from the Alexander von Humboldt Foundation, a Leverhulme Early Career Fellowship, and support from the Isaac Newton Trust. C. M. acknowledges an Oppenheimer Research Fellowship from the School of Physical Sciences, University of Cambridge. This project has received funding from the European Research Council (ERC) under the European Union's Horizon 2020 research and innovation programme (grant agreement no. 714581). This work used the ARCHER UK National Supercomputing Service *via* EPSRC Grant EP/K014560/1. *Data access statement*: original data supporting this publication are available as Electronic Supplementary Information (ESI†).

## Conflicts of interest

There are no conflicts to declare.

## Supplementary Material

Supplementary information

Supplementary information
