# Supplementary material for: Towards an atomistic understanding of disordered carbon electrode materials
Source: Chem Commun (Camb). 2018 May 23;54(47):5988–91. doi: 10.1039/c8cc01388h (PMC5994876; doi:10.1039/c8cc01388h)
Supplement: Supplementary file 2 [file CC-054-C8CC01388H-s002.pdf]

Electronic Supplementary Material (ESI) for ChemComm.
This journal is © The Royal Society of Chemistry 2018

Electronic Supplementary Information (ESI) for

Towards an atomistic understanding of disordered carbon electrode materials

Volker L. Deringer, Céline Merlet, Yuchen Hu, Tae Hoon Lee, John A. Kattirtzi, Oliver Pecher, Gábor Csányi, Stephen R. Elliott, and Clare P. Grey

This ESI document contains:

Supplementary discussion (I): PDF data **S2**

Supplementary discussion (II): Pore sizes **S5**

Supplementary discussion (III): Electronic DOS of pure carbon structures **S6**

Supplementary discussion (IV): Na intercalation from DFT-MD **S8**

Computational details **S10**

Structural details **S12**

Supplementary references **S14**

Supplementary discussion (I): PDF data

Figure 2c of our Communication shows PDF data for a GAP-generated porous carbon structure and for two representative experimental samples. It is stated in the main text that our simulation "reproduces all general features". Here, we discuss in more detail the remaining deviations between computed and experimental results.

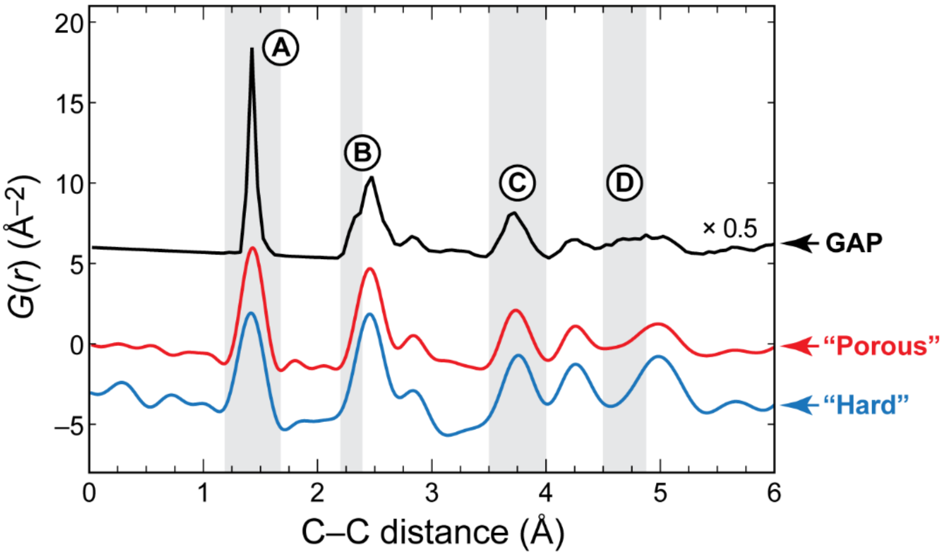

Fig. S1 *(as supplement to Fig. 2c in the main text)*. From top to bottom: Pair distribution functions for a porous carbon structure (scaled down arbitrarily by dividing by a scaling factor of 2), for a carbide-derived carbon from ref S1(shown in Fig. 1d and labelled "TiC-CDC-600" there), and for a hard carbon from ref S2 (shown in Fig. 1a and labelled "pristine" there). Vertical offsets of +6 (–3) $Å^{-2}$ for the GAP (hard carbon) data are chosen to ease visualization, respectively.

There are four main differences between simulated and experimental PDFs. These differences are highlighted by shading in **Fig. S1**:

- The first peak (**A**) is less broad in the GAP data than in both experiments. Our calculated value refers to a single structure (from an MD simulation at 300 K), but has otherwise not been thermally broadened. There are two possible routes by which one could do so. We calculated an averaged PDF for ten different structures from a short (1,000 steps ≡ 1 ps) MD trajectory, but this led to only insignificant changes. We also investigated the effect of artificially adding broadening, by convoluting the simulated PDF data with Gaussians of different widths (0.11 Å, as suggested in Ref. S3, as well as smaller values). The latter procedure, however, led to loss of features: for example,

the previously well-defined peak at 2.8 Å was smoothed out to appear like a shoulder of the peak at 2.5 Å only. We therefore refrain from applying Gaussian broadening to our calculated data.

Some previously presented computational results, obtained with classical interatomic potentials, do show broader distributions in the first PDF peak. We note, however, that these simulations do not follow a quenching trajectory down to 300 K but are quenched only to higher temperatures. This precludes a direct comparison to our data. The quench approach we are using is not a mimetic one, as it here serves only to generate an initial amorphous precursor (in the spirit of commonly used modelling approaches such as in Ref. S3). Other structure-generation strategies might be interesting to explore in the future, and could likewise be coupled to the GAP framework.

- The overall magnitude of the first peak (**A**) is visibly larger in the GAP data than in experiments (recall that scaling has been applied for plotting): numerical integration yields ≈ 1.98 for the GAP structure, compared to ≈ 1.15 for the experimental "porous" carbon (TiC-CDC). To rule out computational artifacts, we integrated over the first peak to obtain the coordination number (CN), which gave 3.25, close to the value of 3.0 in ideal graphene and graphite. For the TiC-CDC, the same procedure yields a CN close to 2 when assuming a density of 1 g cm^{-3}. Note, however, that densities for these experimental samples are difficult to determine, and might be underestimated here (leading to an underestimation of the CN). To account for higher sample densities, we also analysed the PDF for a GAP structure at ≈ 1.5 g cm^{-3}, which led to similar conclusions as the ≈ 1.0 g cm^{-3} one.

- A shoulder on the low-*r* side of the second peak is seen in the GAP data (**B**) but not in experiments. This stems from the presence of 5-membered rings, as clearly identified by our analysis in Fig. 2d. We re-iterate that the count of 5-/7-membered rings in our structures is high, exceeding that in more graphite-like structures, but in turn allowing us to sample diverse local environments. Future work will deal with likewise more ordered GAP-derived structural models.

- The magnitude of the peak at 3.8 Å is somewhat higher in the GAP data (**C**) than in experiments. We believe that a similar argument might hold as made for the higher first peak (**A**). This signal has been linked to one of the cross-ring distances in two adjacent 6-membered rings,[S1] so an overestimation as in case **B** is unlikely to occur here.

- Additional contributions are seen in the computed data around 4.7 Å (**D**), whereas no such signals appear for the experimental samples. Although it is difficult to make a final statement due to the disordered nature of our structures and the (after all) limited sample size, we did inspect interatomic distances (**Fig. S2**), and found several occurrences corresponding to the distance region highlighted as (**D**) in which odd-membered rings are involved. We therefore believe that this region of the PDF can serve as another "fingerprint" for disorder in carbon networks, and that these contributions there will diminish upon further annealing. This will be investigated in future work.

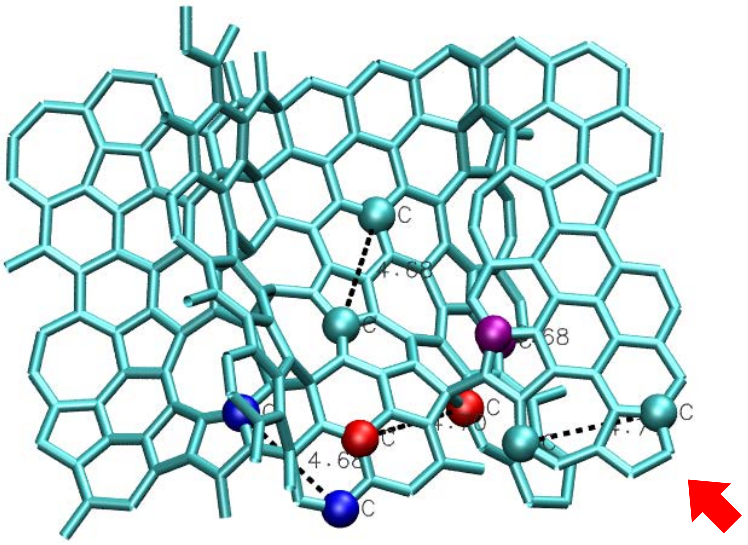

Fig. S2. Structural fragment from a GAP-generated carbon structure, emphasizing interatomic distances that correspond to the additional PDF signals highlighted as (**D**) in Fig. S1. Pairs of atoms are colour-coded individually, and it is seen that several of them involve structural defects or 5-/7-membered rings (one pair of such odd-membered rings is highlighted by an arrow).

Supplementary discussion (II): Pore sizes

To gain further insight into the nature of the pores in our low-density structural models, we used the POREBLAZER 3.0.2 software[S4] (with default parameters) to calculate geometric pore size distributions. In brief, this is realised by using a two-steps Monte Carlo procedure. In the first step, a random point **a** is chosen in a fine 3D grid of points which divides the simulation cell into bins. The program checks for overlaps with the neighbouring atoms considering a van der Waals diameter of 3.431 A for the carbon. If point **a** is not overlapping with the solid, a second Monte Carlo step is done to find the largest sphere that encompasses point **a** and does not overlap with the carbon structure. This process is repeated many times and the cumulative pore volume function $V_p(r)$, i.e. the void space that can be covered by spheres of diameter r or smaller, is built. The geometric pore size distribution is then obtained by differentiating $V_p(r)$. Details of the approach and references to further literature are in Ref. S4.

Results for the small structures (≈ 200 atoms) and a density of ≈ 1 g cm^{-3} are shown in **Fig. S3** as the most representative case. Most of these structures have a single pore occupying a large part of the cell (the precise volume being dependent both on the carbon network and on the optimised mass density); a few structures show a bimodal distribution. The observed pore sizes range from 0.8 to 1.3 nm which is close to what is observed experimentally for TiC-CDC samples of similar densities.[S1,5,6] The generated structures thus provide a range of models compatible with the experimental results, both in terms of local structure (Fig. 2 and S1) and porosity. Nevertheless, it is important to point out that a single small structure (at the size of what is accessible to DFT simulations), having a limited number of pores or even one, is insufficient to represent correctly the wider distribution of pores observed experimentally for bulk materials.

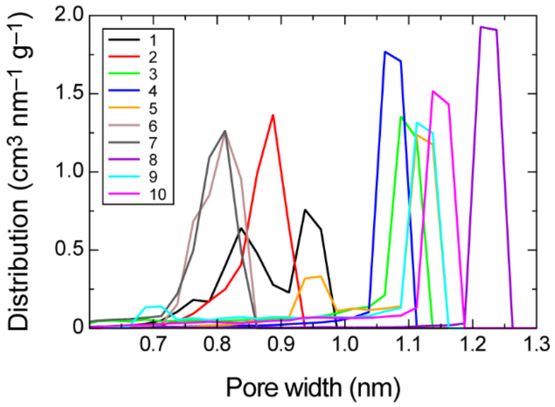

Fig. S3. Pore-size distributions for ten structural models representative of porous carbon, each containing ≈ 200 carbon atoms at a sample density of ≈ 1 g cm^{-3} (numbered as 1–10), as determined using POREBLAZER 3.0.2.[S4]

Supplementary discussion (III): Electronic DOS of pure carbon structures

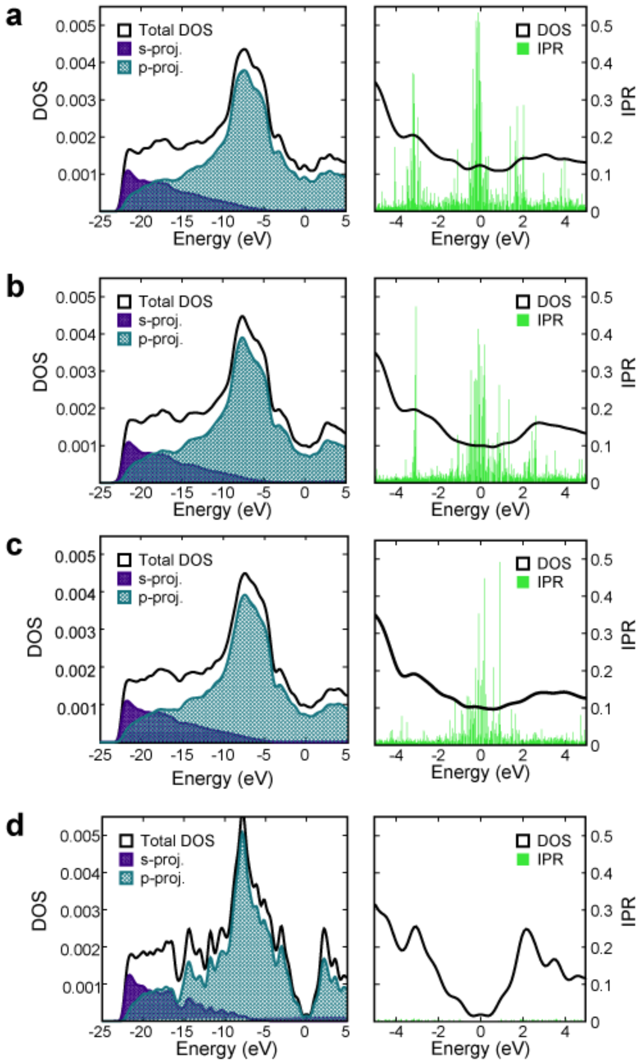

Fig. S4. Electronic structure of disordered carbon systems, generated in GAP-MD simulations, at densities of 1.0 g cm^{-3} (a), 1.5 g cm^{-3} (b), 2.0 g cm^{-3} (c) and of idealised graphene for comparison (d). The panels on the left-hand side give total electronic densities of states (with the Fermi level, E_F, set as the energy zero), as well as projections onto the s (*blue*) and p (*teal*) valence orbitals. The panels on the right-hand side show a close-up of the energy window around E_F, and additionally the inverse participation ratio (IPR) for each Kohn–Sham eigenvalue is given (*green*): larger values indicate localised states, whereas low values indicate higher delocalisation. The IPR around E_F is notable for the disordered systems (panels a–c) but vanishingly small for the idealised graphene system (panel d).

Having access to optimised carbonaceous structures allows us to feed these into DFT computations. In the main text, we focus on an exemplary study of Na intercalation (Fig. 3–4), but the pure frameworks and their electronic structure are amenable to (single-point) DFT analyses as well. Other than in the main text, we are here interested in the *band gap* of the pure structures and therefore employ a more computationally expensive, higher-level DFT method for this particular purpose.

As another example, and to illustrate the usefulness of the approach, we here investigate the electronic structures of systems containing ≈ 1,000 atoms at densities around 1.0 g cm^{-3}, 1.5 g cm^{-3} and 2.0 g cm^{-3}, respectively; for comparison, we study a similarly sized idealised graphene system as well (**Fig. S4**). These computations were performed using CP2K and the hybrid HSE06 functional,[S7,8] which is known to provide accurate electronic band gaps in solids and has been applied to smaller amorphous carbon structural models in a recent study.[S9]

Overall, we find that, as the density increases, and the atomic structure increases in likeness to graphene (*cf.* Fig. 1 in the main text), the electronic structure tends toward graphene too. All porous carbon structures exhibit a computed band gap of zero eV, and the states near the Fermi level, E_F, correspond to p-orbital contributions (Fig. S4a–c), in line with the simplified notion of π systems that do not hybridise with the valence 2s orbitals. The total DOS of graphene (Fig. S4d) has a sharp drop-off near E_F, and the remaining finite DOS is merely a consequence of the Γ-point sampling and Gaussian broadening applied to the electronic levels. As the density of the amorphous structures increases from 1.0 to 2.0 g cm^{-3}, the absolute DOS at E_F decreases by approximately two thirds, again emphasizing the closer correspondence to idealised graphene.

The states near E_F are further analysed using the Inverse Participation Ratio (IPR), which quantifies the localization of the Kohn-Sham eigenstates and is inversely proportional to the number of atoms on which a given eigenstate is localised. The number of states with an IPR value > 0.2 in the 5 eV range below E_F decreases from 15 to 10 to 2 as the density increases from 1.0 to 1.5 to 2.0 g cm^{-3}. Visual inspection of the wave functions revealed that localised states near E_F are mainly localised on regions of disorder, such as odd-membered rings or undercoordinated sites.

Amorphous porous carbons are used as electrodes in supercapacitors, as ions can enter the pores. When applying a voltage to the electrodes, a high electronic conductivity is desirable; however, theoretical and experimental work has shown that the conductivity of amorphous carbons is much lower than that of graphene.[S10–12] The localised states near E_F seen in Fig. S4 indicate that electron hopping between localised states is required. As the localisation near E_F decreases with increasing carbon density, the electronic conductivity is likely to increase.

Supplementary discussion (IV): Na intercalation from DFT-MD

As stated in the main text, we performed DFT-MD simulations (annealing at 1,000 K for 10 ps and subsequent cooling to 10 K) for the same carbon structure but with different numbers of intercalated Na atoms. **Figure S5** provides an overview of the resulting structures, with an emphasis on the behaviour of Na atoms. In the case of low filling (a), the atoms are isolated, consistent with the notion of positively charged "Na^+" ions repelling each other. On increasing the concentration of Na atoms slightly (b), we observe the formation of a five-membered cluster at the endpoint of the MD trajectory, whereby we define a "cluster" through connectivity, drawing bonds up to a maximum Na–Na distance of 3.8 Å. A further increase of the Na concentration leads to the formation of a larger, nine-atom cluster in the large pore of the same host structure (and only there). Figure 4 in the main text shows how the occurrence of such Na–Na contacts and, ultimately, clustering, is concomitant with lowered charges on the atoms involved: that is, with a gradual back-transfer of electrons and a transition from "Na^+" to "Na^0" (see discussion in the main text).

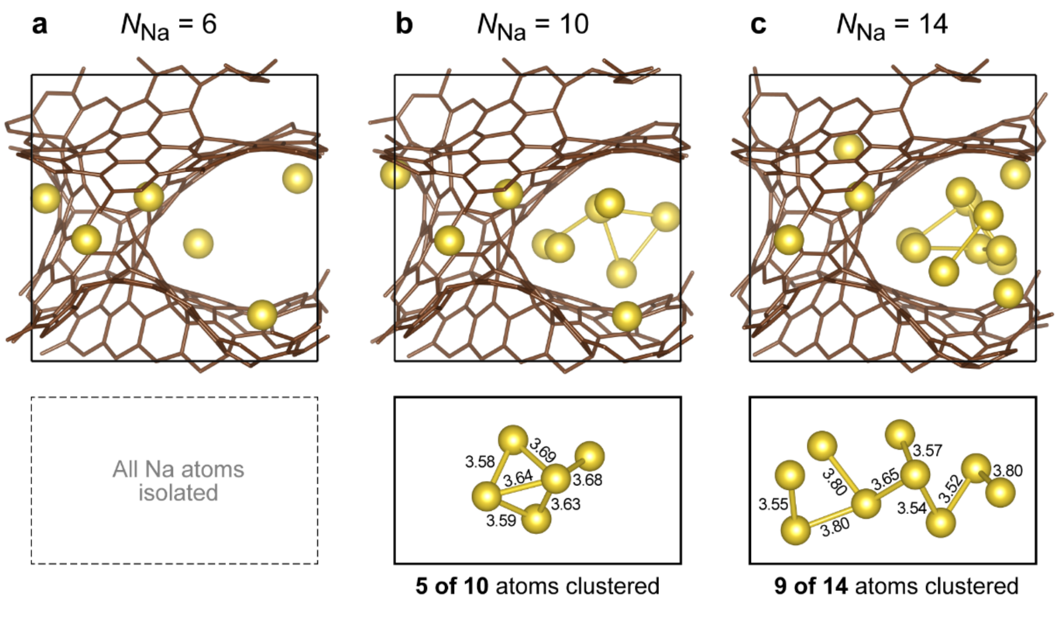

Fig. S5. Structural snapshots after DFT-MD annealing and cooling, in parallel runs, for a carbon framework with different numbers, N_{Na}, of intercalated atoms. The upper panels show the entire structure (carbon atoms as wireframe, Na atoms as yellow spheres; viewed down the *a*-axis of the simulation cell). The lower panels show close-ups of the resulting clusters, with Na–Na distances given in Å. We draw bonds between Na atoms with a spacing of 3.8 Å or closer.

It is stated in the main text that the Na filling and high-temperature MD simulations leave the carbon structure "largely unaffected" (p. 3). To support this statement, we provide here structural drawings of the same carbon framework in different scenarios, omitting Na atoms for clarity (**Fig. S6**). From left to right, we show

i. an exemplary, DFT-relaxed structure resulting from the *ab initio* random structure searching (AIRSS; Ref. 43 in the main text) like procedure used in Fig. 3 (insertion of a single Na);
ii. a structural snapshot after 5,000 timesteps (5 ps) of high-temperature (1,000 K) DFT-MD annealing of an $Na_{14}C_{206}$ structure (*i.e.*, a case with much higher filling);
iii. the same after 10,000 timesteps (10 ps), indicating the stability over time of the carbon framework;
iv. the same structure after cooling in MD, which allows the Na atoms to settle into their local minima.

Two different view directions (top, down the *a*-axis; bottom, down the *b*-axis) are shown and both confirm that there are no significant changes in the connectivity, or in the overall structure, of the carbon framework during our simulations.

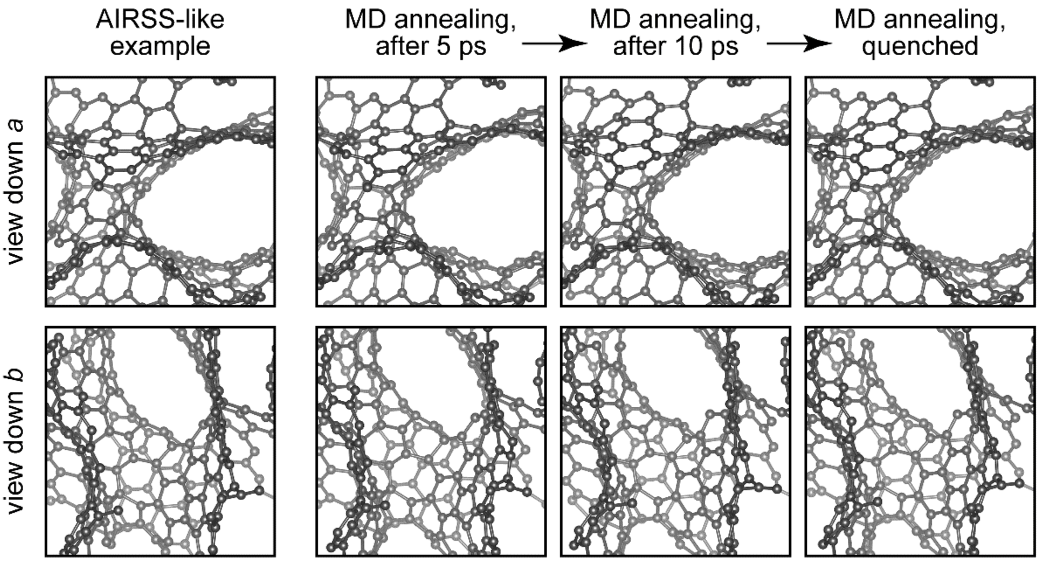

Fig. S6. Structural drawings of a porous carbon structural model with different degrees of Na filling, either in AIRSS-like DFT relaxations (one Na atom in the cell) or at various stages of a DFT-MD annealing and cooling trajectory (14 Na atoms in the cell), **showing here only the carbon atoms** for direct comparison.

Computational details

Partial ring analysis. The contributions from different ring types (as shown in Fig. 2d in the main text) were analysed for internal distances only (*i.e.*, distances inside the rings). The R.I.N.G.S software[S13] was used to identify all 5-, 6- and 7-membered rings in the GAP-generated structures and to provide lists of the carbon atoms belonging to these different rings. This output was then used to calculate carbon–carbon distances for each of the lists, thus separating the PDF contributions from 5-, 6- and 7-membered rings. We could check easily that the sum of all these contributions is equal to the total PDF calculated in the usual way.

GAP modelling. The QUIP / quippy code, which is freely available for non-commercial research at http://www.libatoms.org, was used for all GAP-driven MD simulations. Amorphous precursors were generated by rapid quenching from the melt as described in our previous publication[S14] and in line with established procedures in the community.[S15] The following protocol was then used to generate porous and partly graphitised structures:

- **Heat to 3,000 K** over 10 ps, with a stepwise increase in temperature every 1 ps;
- **Anneal at 3,000 K** for 100 ps;
- "**Prune**" the resulting structure of unfavourable fragments:
  - identify all atoms with a CN of < 2 ("dangling-bond defects") and
  - identify all atoms with CN = 2 whose both neighbours similarly have CN = 2 (atoms in close-to-linear "sp-like" chains); remove all atoms so identified;
  - repeat the above steps until no unfavourable fragments remain in the structure.
- Anneal at 3,000 K for a further 100 ps; prune as above; anneal for a final 100 ps;
- **Cool to 300 K** over 10 ps, with a stepwise lowering of the temperature every 1 ps;
- **Optimise the resulting structure**, using the following protocol:
  - create five copies of the cell with the lattice parameters scaled by –2%, –1%, 0%, +1%, +2%, respectively; for each of these copies
  - perform an MD simulation with exponential quench from 300 K to 10 K over 5 ps;
  - then optimise atomic positions using the LBFGS algorithm;[S16]
  - fit the resulting $E(V)$ data to the Birch–Murnaghan equation of state;[S17]
  - perform a final MD quench and optimization as above at the so-determined optimum unit-cell volume.

The timestep was 1 fs in all simulations.

The above protocol was carried out for initial densities of 1.0, 1.25, 1.5, 1.75, and 2.0 g cm^{-3}, respectively; the final densities varied slightly from sample to sample, due to the removal of atoms in some cases and the optimization of the unit cell; results are given below. We studied 10 systems in parallel at an initial system size of 216 atoms/cell, giving rise to 50 candidate structures, which were subjected to DFT computations as below. We furthermore created 3 systems with 1,000 atoms in the cell and initial density settings of 1.0, 1.5, and 2.0 g cm^{-3}, using the same GAP annealing and optimization procedure but not subjecting these to further DFT optimization, due to the large cost of performing structural relaxations at this system size.

Among the 5 × 10 = 50 "small" structural models (≈ 200 atoms/cell), four showed the formation of unfavourable chain motifs after optimization, and these were therefore excluded. One of the partly graphitised samples was used in a different, methodological study by some of us very recently (Ref. 35), and is therefore excluded from the dataset presented and analysed here. The remaining 45 structures are further characterised below, and provided as Supporting Information in ZIP format, together with the 3 large structural models. We stress again that the annealing of amorphous samples into graphitised carbons is well-established in the community;[S3,18,19] the novelty of the work here is in the link to energy materials, in the study of Na intercalation, and in the connection with DFT post-processing to further understand experimental observations.

DFT relaxation. DFT computations were carried out using the projector augmented-wave (PAW) method[S20] as implemented in VASP.[S21–24] The plane-wave energy cut-off was 500 eV, the SCF halting criterion was $\Delta E < 10^{-6}$ eV, and reciprocal space was sampled at Γ. The PBE functional[S25] together with pairwise Tkatchenko–Scheffler dispersion corrections[S26,27] was used for structural optimisations, such as to minimise residual forces on atoms below 0.01 eV $Å^{-1}$.

Single-point energies. It is known that many-body dispersion (MBD) effects, beyond pairwise additive terms, play an important role in carbon nanostructures.[S28] We therefore computed single-point energies for the optimised pure carbon structures using the MBD approach by Tkatchenko and co-workers,[S29–31] as implemented in VASP by Bučko and co-workers.[S27,32] In contrast, intercalation energies for single Na atoms (translated into voltages), were computed at the PBE level, as these pertain to *relative* energy differences between two largely similar carbon networks and to the Na–C interaction instead.

Structural details

Due to the large file sizes, all structures are provided as a separate ZIP file (available as ESI), containing POSCAR (VASP input) as well as XYZ format.

Relevant details of the generated 216-atom structures are given in **Table S1** below, namely:

a) the **initial setting for sample density** (at which the amorphous precursor was generated);
b) a **running index** (as used in the attached ZIP file to identify individual structures);
c) the **“optimised” density**, that is, after removal of defects during annealing and subsequent structural optimization (including optimization of the cell volume);
d) the **total VASP-computed energy** at the PBE+MBD level (see Computational details section above);
e) the **relative energy**, given as the difference from the most stable structure found, and per atom;
f) a qualitative identification of the **structure type** (based on visual inspection), labelling each entry as “porous” (P) structures containing voids, mainly found at lower density, or “graphite-like” (G) showing extended defective 2D sheets, mainly found at higher density (see Fig. 1 in the main text for an exemplary illustration).

Table S1. Overview of ≈200-atom structures generated in this work (see text for details).

**Initial density** (g cm^{-3})	**Running index**	**Optimised density** (g cm^{-3})	**Total MBD energy** (eV/cell)	**Relative MBD energy** (eV/cell)	**Structure type**
1.25	9	1.26	–1934.366255	0.23	P
1.00	5	0.89	–1703.354218	0.22	P
1.50	7	1.55	–1938.198988	0.21	P
1.00	6	0.91	–1705.213146	0.21	P
1.00	3	1.00	–1849.304343	0.21	P
1.25	7	1.20	–1859.520649	0.20	P
1.50	4	1.51	–1941.210675	0.20	P
1.75	10	1.63	–1869.523066	0.20	P
1.50	8	1.40	–1852.268671	0.19	P
1.25	1	1.19	–1835.022833	0.19	P
1.25	2	1.03	–1682.358214	0.19	P
1.25	3	1.25	–1891.111241	0.18	G
1.00	7	1.01	–1918.199375	0.18	P
1.50	5	1.41	–1864.247264	0.18	G
1.75	4	1.65	–1919.607073	0.17	P
1.50	9	1.44	–1814.105243	0.16	G
1.00	1	0.96	–1870.032298	0.15	P
1.00	2	0.91	–1771.151839	0.15	P
1.00	4	0.89	–1707.934429	0.15	P
2.00	2	2.01	–1951.964369	0.15	G
1.00	9	0.95	–1927.360101	0.14	P
2.00	3	2.01	–1954.592634	0.14	G
2.00	6	2.10	–1955.222882	0.13	G
1.75	3	1.78	–1901.372544	0.13	G
1.25	5	1.27	–1902.255573	0.13	G
1.00	8	0.81	–1558.117307	0.13	P
1.25	4	1.17	–1884.721183	0.12	P
1.75	5	1.84	–1957.327330	0.12	G
1.75	1	1.73	–1922.035746	0.12	G
1.50	3	1.52	–1959.742225	0.11	G
1.50	1	1.34	–1789.149915	0.10	G
1.50	6	1.36	–1844.852374	0.10	G
1.75	2	1.83	–1926.993346	0.10	G
2.00	8	2.03	–1963.952309	0.09	G
1.25	8	1.16	–1965.250192	0.09	G
2.00	4	1.92	–1965.865238	0.08	G
1.75	6	1.55	–1847.805519	0.08	G
1.25	10	1.26	–1967.329593	0.08	G
2.00	9	2.08	–1967.423880	0.08	G
1.75	9	1.81	–1895.458806	0.07	G
2.00	5	2.06	–1968.826988	0.07	G
1.75	7	1.69	–1942.024015	0.07	G
2.00	7	1.92	–1969.636874	0.07	G
1.75	8	1.72	–1970.413127	0.06	G
2.00	10	1.75	–1836.942497	0.00	G

Supplementary references

1 A. C. Forse, C. Merlet, P. K. Allan, E. K. Humphreys, J. M. Griffin, M. Aslan, M. Zeiger, V. Presser, Y. Gogotsi and C. P. Grey, New Insights into the Structure of Nanoporous Carbons from NMR, Raman, and Pair Distribution Function Analysis, *Chem. Mater.*, 2015, **27**, 6848–6857.

2 J. M. Stratford, P. K. Allan, O. Pecher, P. A. Chater and C. P. Grey, Mechanistic insights into sodium storage in hard carbon anodes using local structure probes, *Chem. Commun.*, 2016, **52**, 12430–12433.

3 C. de Tomas, I. Suarez-Martinez, F. Vallejos-Burgos, M. J. López, K. Kaneko and N. A. Marks, Structural prediction of graphitization and porosity in carbide-derived carbons, *Carbon*, 2017, **119**, 1–9.

4 L. Sarkisov and A. Harrison, Computational structure characterisation tools in application to ordered and disordered porous materials, *Mol. Sim.*, 2011, **37**, 1248–1257.

5 R. Dash, J. Chmiola, G. Yushin, Y. Gogotsi, G. Laudisio, J. Singer, J. Fischer and S. Kucheyev, Titanium carbide derived nanoporous carbon for energy-related applications, *Carbon*, 2006, **44**, 2489–2497.

6 L. Borchardt, M. Oschatz, S. Paasch, S. Kaskel and E. Brunner, Interaction of electrolyte molecules with carbon materials of well-defined porosity: characterization by solid-state NMR spectroscopy, *Phys. Chem. Chem. Phys.*, 2013, **15**, 15177.

7 J. Heyd, G. E. Scuseria and M. Ernzerhof, Hybrid functionals based on a screened Coulomb potential, *J. Chem. Phys.*, 2003, **118**, 8207–8215.

8 J. Heyd, G. E. Scuseria and M. Ernzerhof, Erratum: 'Hybrid functionals based on a screened Coulomb potential' [J. Chem. Phys. 118, 8207 (2003)], *J. Chem. Phys.*, 2006, **124**, 219906.

9 F. Risplendi, M. Bernardi, G. Cicero and J. C. Grossman, Structure-property relations in amorphous carbon for photovoltaics, *Appl. Phys. Lett.*, 2014, **105**, 43903.

10 J. Robertson, Amorphous carbon, *Adv. Phys.*, 1986, **35**, 317–374.

11 C. Godet, Hopping model for charge transport in amorphous carbon, *Philos. Mag. B*, 2001, **81**, 205–222.

12 L. J. Kennedy, J. J. Vijaya and G. Sekaran, Electrical conductivity study of porous carbon composite derived from rice husk, *Mater. Chem. Phys.*, 2005, **91**, 471–476.

13 S. Le Roux and P. Jund, Ring statistics analysis of topological networks: New approach and application to amorphous GeS_2 and SiO_2 systems, *Comput. Mater. Sci.*, 2010, **49**, 70–83.

14 V. L. Deringer and G. Csányi, Machine learning based interatomic potential for amorphous carbon, *Phys. Rev. B*, 2017, **95**, 94203.

15 N. A. Marks, in *Computer-Based Modeling of Novel Carbon Systems and Their Properties: Beyond Nanotubes*, eds. L. Colombo and A. Fasolino, Springer, Dordrecht, 2010, pp. 129–169.

16 D. C. Liu and J. Nocedal, On the limited memory BFGS method for large scale optimization, *Math. Program.*, 1989, **45**, 503–528.

17 F. Birch, Finite Elastic Strain of Cubic Crystals, *Phys. Rev.*, 1947, **71**, 809–824.

18 R. C. Powles, N. A. Marks and D. W. M. Lau, Self-assembly of sp2-bonded carbon nanostructures from amorphous precursors, *Phys. Rev. B*, 2009, **79**, 75430.

19 C. de Tomas, I. Suarez-Martinez and N. A. Marks, Graphitization of amorphous carbons: A comparative study of interatomic potentials, *Carbon*, 2016, **109**, 681–693.

20 P. E. Blöchl, Projector augmented-wave method, *Phys. Rev. B*, 1994, **50**, 17953–17979.

21 G. Kresse and J. Hafner, Ab initio molecular dynamics for liquid metals, *Phys. Rev. B*, 1993, **47**, 558–561.

22 G. Kresse and J. Furthmüller, Efficiency of ab-initio total energy calculations for metals and semiconductors using a plane-wave basis set, *Comput. Mater. Sci.*, 1996, **6**, 15–50.

23 G. Kresse and J. Furthmüller, Efficient Iterative Schemes for Ab Initio Total-Energy Calculations Using a Plane-Wave Basis Set, *Phys. Rev. B*, 1996, **54**, 11169–11186.

24 G. Kresse and D. Joubert, From ultrasoft pseudopotentials to the projector augmented-wave method, *Phys. Rev. B*, 1999, **59**, 1758–1775.

25 J. P. Perdew, K. Burke and M. Ernzerhof, Generalized Gradient Approximation Made Simple, *Phys. Rev. Lett.*, 1996, **77**, 3865–3868.

26 A. Tkatchenko and M. Scheffler, Accurate Molecular Van Der Waals Interactions from Ground-State Electron Density and Free-Atom Reference Data, *Phys. Rev. Lett.*, 2009, **102**, 73005.

27 T. Bučko, S. Lebègue, J. Hafner and J. G. Ángyán, Tkatchenko-Scheffler van der Waals correction method with and without self-consistent screening applied to solids, *Phys. Rev. B*, 2013, **87**, 64110.

28 V. V Gobre and A. Tkatchenko, Scaling laws for van der Waals interactions in nanostructured materials, *Nat. Commun.*, 2013, **4**, 2341.

29 A. Tkatchenko, R. A. DiStasio, R. Car and M. Scheffler, Accurate and efficient method for many-body van der Waals interactions., *Phys. Rev. Lett.*, 2012, **108**, 236402.

30 A. Ambrosetti, A. M. Reilly, R. A. DiStasio and A. Tkatchenko, Long-range correlation energy calculated from coupled atomic response functions., *J. Chem. Phys.*, 2014, **140**, 18A508.

31 R. A. DiStasio, V. V Gobre and A. Tkatchenko, Many-body van der Waals interactions in molecules and condensed matter., *J. Phys. Condens. Matter*, 2014, **26**, 213202.

32 T. Bučko, S. Lebègue, T. Gould and J. G. Ángyán, Many-body dispersion corrections for periodic systems: an efficient reciprocal space implementation., *J. Phys. Condens. Matter*, 2016, **28**, 45201.
